# The History of Makassan Trepang Fishing and Trade

**DOI:** 10.1371/journal.pone.0011346

**Published:** 2010-06-29

**Authors:** Kathleen Schwerdtner Máñez, Sebastian C. A. Ferse

**Affiliations:** Leibniz-Center for Tropical Marine Ecology (ZMT), Bremen, Germany; University of Canterbury, New Zealand

## Abstract

The Malayan term *trepang* describes a variety of edible holothurians commonly known as sea cucumbers. Although found in temperate and tropical marine waters all over the world, the centre of species diversity and abundance are the shallow coastal waters of Island Southeast Asia. For at least 300 years, trepang has been a highly priced commodity in the Chinese market. Originally, its fishing and trade was a specialized business, centred on the town of Makassar in South Sulawesi (Indonesia). The rise of trepang fishing in the 17^th^ century added valuable export merchandize to the rich shallow seas surrounding the islands of Southeast Asia. This enabled local communities to become part of large trading networks and greatly supported their economic development. In this article, we follow Makassan trepang fishing and trading from its beginning until the industrialization of the fishery and worldwide depletion of sea cucumbers in the 20^th^ century. Thereby, we identify a number of characteristics which trepang fishing shares with the exploitation of other marine resources, including (1) a strong influence of international markets, (2) the role of patron-client relationships which heavily influence the resource selection, and (3) the roving-bandit-syndrome, where fishermen exploit local stocks of valuable resources until they are depleted, and then move to another area. We suggest that understanding the similarities and differences between historical and recent exploitation of marine resources is an important step towards effective management solutions.

## Introduction

*Living in a trader's house everything is brought to me as well as to the rest–bundles of smoked tripang, or beche de mer, looking like sausages which have been rolled in mud and then thrown up the chimney… Alfred Russel Wallace [1:329]*

The term trepang derives from the Malayan word *teripang* and describes a range of edible holothurians commonly known as sea cucumbers. Sea cucumbers are found in temperate and tropical marine waters all over the world, but the centre of species diversity and abundance are the shallow coastal waters of Island Southeast Asia and adjacent areas. In this region, some 80–100 species are known, up to half of which have some commercial value. Along with other marine resources such as pearls, mother-of-pearl and tortoiseshells, trepang has exclusively been harvested as a high-priced commodity for the international market. Historically, trepang trade was a specialized business, almost completely in the hand of Makassarese, Bugis, and Bajau [2]. Because of its importance as an item of trade, and certainly also because of its unusual appearance to early European travellers, there are a number of historical sources available allowing us to follow the history of trepang fishing and trade in Island Southeast Asia, with Makassar (Ujung Pandang) in South Sulawesi as a major trading hub.

The consumption of trepang is almost entirely restricted to the Chinese, who consider them a culinary delicacy and an aphrodisiac. Together with similarly treasured resources such as shark fins and bird nests, trepang belongs to a group of commodities which were so valuable that “distant seaside cliffs and seemingly peripheral seas became sought-after destinations” [3:138]. Trepang fishing and trade thus was exclusively driven by a strong demand from China, and quickly generated new sources of supply. Trade with China has a long history in Southeast Asia, reaching back several thousand years and resulting in the development of large trading networks that involved local partners and extended into remote areas such as Eastern Indonesia by the time the trepang fishery developed [4]. The shallow seas of Island Southeast Asia suddenly had an additional valuable export commodity enabling local communities to contribute to these networks.

Authors such as Macknight [5], Sutherland [3] and Dai [6] agree that sea cucumbers were first derived from Hainan and Japan, before Southeast Asia became the centre of exploitation. The region not only possessed what seemed like an unlimited supply, it also offered virtually ideal conditions for exploitation and trade.

Makassar had long been known as a central point of seaborne commerce. Its geographic location at the southwest peninsular of Sulawesi made the town an ideal trading place, being at the crossroads of local coastal movements as well as in inter-insular traffic among Java, Kalimantan, Maluku, Nusa Tenggara and the Philippines, and in long distance trade with Europe, India and China [7]. The town also provided political stability, which further increased after its conquest by the Dutch in 1669. In addition, many Indians and Malays withdrew from Makassar after 1669, thereby leaving room for Chinese traders [3].

Three ethnic groups were involved in the exploitation and trade of marine resources in this region. The Bajau people originally were sea nomads, travelling long distances in search of valuable collecting grounds and thereby opening many trading routes. Bajau have often been described as the only people engaged in commercial fishing and collecting resources along the shores in Sulawesi [8]. They added trepang catching to fishing and turtle hunting as a means of obtaining goods for exchange [7]. The second group are the Bugis, traders from the mainland of Sulawesi, which Earl [9:390] described as “… the chief and almost sole carriers of the Archipelago, collecting the products of the various islands….” Bugis were also well-known and feared as pirates, and involved in the slave trade [10]–[11]. Together with the Makassarese, the traditional inhabitants of coastal Southern Sulawesi and also well-known as traders, the Bugis became quickly involved in the trepang trade.

Similar to other products, trepang flowed through a shifting hierarchy of collecting points where cargoes were assembled. Many Bugis traders paid dues to their kings or patrons, who could also be their creditors, while the Bajau were often tied to a patron in semi-tributary relationships [3].

Gathering trepang neither required special skills nor a lot of equipment. Fishing techniques ranged from simply collecting specimens by hand to the use of single- or double-headed spears. In shallow water, trepang was located by feeling for it with bare feet and then brought to the surface. Women would also collect the specimens by hand on the reef flats at low tide, while men dived or used a weighted, three-pronged spear, which was lowered by rope from a boat to a point just above a trepang and then dropped to the bottom, the weighted spear impaling the animal [12]. The subsequent processing required more attention, as this contemporary description explains: “After the trepang is caught, it is immediately boiled in sea-water, in which the leaves of the papaya are steeped, to take off a thin skin which covers it. It is then placed in baskets or holes, and covered up with earth until the following morning, when it is washed repeatedly to deprive it as much as possible of the disagreeable taste of coral which it possesses, after which it is spread out on mats, and dried.” [13:174]. Sometimes, instead of papaya, mangrove bark was used, and in some areas the trepang was also dried by smoking over fire. In this way made to last, the trepang was either sold to the Chinese in Makassar, or directly brought to Singapore [10]. The mode of production has not changed over time. Nowadays, trepang fishers still process their harvest in basically the same way.

Fishing and trading of trepang has a number of similarities with the more recent exploitation of other marine commodities, such as live reef food fish or ornamentals. One major factor common to all is the strong influence of an outside demand. The raise of a wealthy consumer class not only had a great influence on the Chinese desire for sea cucumbers in the late seventeenth and early eighteenth century [14], but also stimulates today's exports of live grouper to seafood restaurants in Hong Kong and Singapore. Similarly, fashionable trends greatly influence the demand for marine commodities: the movie “Finding Nemo” significantly increased the demand for both saltwater aquariums and clownfishes.

A second influential factor is the role of credit and debt, which is inherent in patron-client relationships. The term describes a relationship between a politically and economically powerful patron and a weaker client. Clients and their families can borrow money, equipment or goods from the patron, in order to make it through bad seasons. This can be regarded as benevolent, but also creates debts and dependency. Patron-client relationships have a significant influence on the exploitation of marine resources in certain parts of Island Southeast Asia. In South Sulawesi, they developed from (1) local systems of land tenure and agricultural production [15] and (2) credit-debt arrangements common in Chinese business operations. *Punggawa*, the local term for patron, has already been used in the 19^th^ century to describe elected leaders of the Bajau [16]. In the eighteenth and nineteenth century credits were usually provided by ethnic Chinese, as Earl noticed: “Many of the Bajau … are chiefly employed by the Chinese in fishing for trepang … and according to the policy invariable adopted by the latter in their dealings with the natives, are generally involved in debt, from which extrication is nearly hopeless … no instance is on record of ever having absconded to avoid the payment of their debts.” [9:335]. For a more detailed description of the role of credits and depths in the Makassan trepanging activities, readers are referred to Sutherland [17].

Patrons react to market signals such as the desire for a new resource by providing their clients with the necessary equipment and announcing their will to buy a certain item. Clients–depending on the credits provided and often socially tied to a patron–will change to a different target resource, if their patron demands them to do so. Thus, patron-client relationships influence the choice of fishing strategies of individual fishermen [18].

Opportunistic behaviour is also quite common in the exploitation of open-access marine resources. Fishermen exploit local stocks of valuable resources until they are depleted, and then move to another area, a pattern Berkes et al. [19] have termed the “roving bandit syndrome”. This sequential exploitation of resources has been described for a range of marine resources such as lobster and conch, sea urchins, and live reef food and ornamental fishes [20]–[22]. While the phenomenon has been linked to the effects of recent globalisation, the case of trepang shows that roving bandits are not an entirely new phenomenon. Other historical accounts also give evidence of early serial depletion of marine resources, as in the case of the Atlantic cod in the 19^th^ century [23], or very large green turtles before the 19^th^ century in the Americas [24].

A current study by the FAO claims sea cucumber overexploitation to be a recent problem [25]. This statement can only be revised by taking a historical perspective, although the present amount of overfishing seems to largely exceed the historical impacts of this activity. While more ecological studies are needed to understand the potential effects of removing a large number of bioturbating organisms from tropical marine ecosystems, there also is a need to look at the similarities and differences between historical and recent exploitation of trepang in order to provide a knowledge base for its management.

Therefore, the aim of this paper is to follow the trepang fishing and trade as an example for the historical and current exploitation of marine resources in Island Southeast Asia. Our focus is on the trade which was started and is still handled by people from Makassar, and which had historically accounted for the largest amount of this economically important activity.

## Results

### The growth of the trade

While some authors stated that sea cucumbers have been harvested for over 1000 years in the Indo-Pacific region [26 and references therein], documented evidence is only available for the last 400 years. In China, written references to trepang appeared for the first time under its Mandarin name *haishen* (sea ginseng) in a book called *Miscellanies of Five Items* in 1602. It was described as an aphrodisiac. More frequent references began to emerge in the late seventeenth century and indicate that trepang must have already been a common food at this time. According to Chinese literature, trepang was found along most of Chinas coastal areas, but the local production soon failed to meet the demand. It was therefore first imported from Japan, and later from Southeast Asia [6]. Documentary evidence from Island Southeast Asia confirms a time span between the late seventeenth and the early eighteenth century as the date for the rise of trepang trade in this region, which is further supported by the fact that none of the extensive and detailed records of the sixteenth and early seventeenth century's trade mentions it [5]. The earliest Dutch record that mentions trepang is the official diary (*daghregister*) of Makassar from June 1710 that refers to trepang collection off Buton (I) in Southeast Sulawesi [7]. Yet, Boomgard [2:113] concludes that trepang seemingly “appears out of nowhere” in 1720 in the records of the Dutch East India Company (*Vereenigde Oost-Indische Compagnie* or VOC). However, from the mid-eighteenth century on, there are frequent references to the collection and processing of trepang all over Island Southeast Asia, with Makassar in South Sulawesi as the centre of trade.

**Figure 1:**
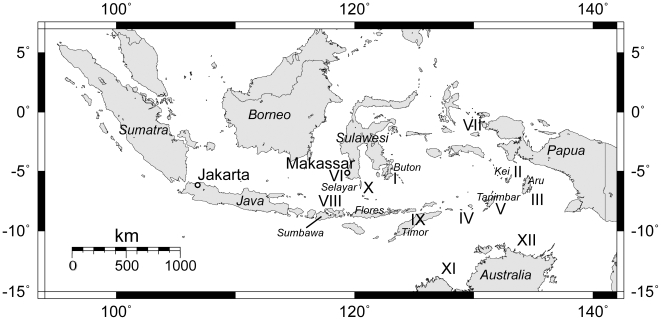
Map of Indonesia, showing important trepang collection areas. The roman numbers refer to passages in the text.

Bugis had probably been visiting the Moluccas–namely the islands of the Kei group (II) –since the sixteenth century [27] (see Figure 1), and there is evidence that trepang fishing in this region actually started around this time. The first hint came from Earl [9], who wrote that the Dutch navigator Pieter Pieterson visited the Aru Islands (III) in 1636 and had reported that the trepang fishery then merely existed. One of the oldest documented sources is a VOC report from 1720, mentioning vessels from Sulawesi and Flores looking for trepang near the islands of Luang and Moa in the Southern Moluccas (IV). Their number was considerable, with 60 and 30 vessels, respectively [2].

Both Kei and Aru soon developed into major fishing grounds. While it seemed that Aru islanders originally sailed to Ambon in order to sell their commodities, after the 1820s traders started to visit people directly in their villages [28]. Bugis, Makassarese, and others brought rice and arak from Makassar and exchanged them for trepang, pearls, tortoise shells and other products. The trade greatly stimulated fishing. Whereas the Aru islanders originally” did not embark on their fishing excursions until after they had received advances from the traders, who had to await their return “… they now fished whenever the weather permitted” whether traders were present or not “[29:492–493]. Kolff [13:175–176]; described the collection: “At low water hundreds of men, with their wives and children, may be perceived wading from Vorkay towards these islets, (the water being only two or three feet deep,) carrying a basket at their backs, and having in their hands a stick provided with an iron point. When the water is deeper than this, they make use of canoes. For fishing on the banks situated at a greater distance, the Arafuras use a prahu, constructed for the purpose, in which they embark their entire family.' From the 1840s, Chinese traders coming from Singapore through Makassar entered the trade networks in Aru and ended the monopoly of the Bugis and Makassarese [30].

The importance of trepang for the livelihoods of many people in the Moluccas is also documented by the fact that institutions which governed the right to capture marine animals were developed [31]. Kolff (1840) reported that the people of Luang in the southern Moluccas claimed the exclusive privilege of fishing trepang in the surrounding waters. He also described that in the islands of Tanimbar (V), the territory of each village consisted of a portion of land and contiguous trepang banks, which were governed by a chief [13]. In the Moluccas, certain marine tenure rights and management responsibilities were part of a culturally embedded institution known as *sasi*. *Sasi* encompasses fishery-related rights and rules, and is therefore commonly referred to as a traditional resource management institution, even though it also has other functions and its focus has changed through time [32]. The term signifies harvest restrictions by temporal closures of collection areas implemented by a local village council, the so-called *kewang*. At least since the 18^th^ century, a specific form of *sasi* was applied to trepang, the *sasi teripang*. Some areas of the Aru Islands employed *sasi teripang* until recently [30]. Obviously, trade and commercialisation supported the establishment of traditional controlled areas [33]. While *sasi* thus is a local institution, it has been suggested that Dutch colonial administrators did assist local elites in recrafting and deploying *sasi* practices to control access to marine commodities [34].

At least since the 1720s, trepang was also fished in South Sulawesi, namely the Spermonde Archipelago (VI) to the north and west of Makassar. Although many of the Spermonde islands were not yet inhabited, some already were settled, such as the island of Barrang Lompo [35] which still is a major trepang trading hub today. Annual imports to Makassar during that time were about 84 *pikul* (1 *pikul* was equivalent to 61.511 kg, the amount a man could carry with a shoulder-pole [36]), a little more than five tons. Most of the trepang came from Barru about 75 km north of Makassar, but significant amounts also arrived from Buton in Southeast Sulawesi, probably a transit point for trepang that really was collected around Aru, Tanimbar and Australia [37]. It is important to keep in mind that these figures are based on registered imports, which only provided a part of Makassar's official exports. A significant amount of trepang probably arrived in unregistered ships, such as small coastal crafts fishing in the rich trepang grounds close to Makassar [7].

In 1717–1718, 11 tons of trepang were exported from Makassar. In the following years, the trepang trade started to grow. In 1725–1726, the amount rose to 79 tons, in 1766–1767 to 301 tons, and in 1787–1788 to 512 tons, respectively (see Table 1). At the end of the eighteenth century, trepang was the most important commodity in Makassar in terms of value, both incoming and outgoing [38].

**Table 1:** Annual trepang exports from Makassar 1717–1917.

Year	Amount in tons	Source
1717–18	11 a	[38]
1722–23	34 a	[38]
1723–24	22 a	[38]
1724–25	31 a	[38]
1725–26	59 a	[38]
1733–34	71 a	[38]
1766–67	301 a	[38]
1767–68	146 a	[38]
1768–69	193 a	[38]
1774–75	292 a	[38]
1775–76	317 a	[38]
1776–77	235 a	[38]
1786–87	399 a	[38]
1787–88	512 a	[38]
1788–89	393 a	[38]
1796–97	154 a	[38]
1820s	∼430 a	[44]
1832	∼300 a	[51]
1833	<134 a	[51]
1834	<134 a	[51]
1868	563	[67]
1869	439	[67]
1870	502	[67]
1871	197	[67]
1872	540	[67]
1873	572	[67]
1874	652	[67]
1875	444	[67]
1876	318	[67]
1877	338	[67]
1878	423	[67]
1915	569	[40]
1916	544	[40]
1917	524	[40]

a In the source, the weight is given in *pikul*, which in the early 19^th^ century equalled 61.511 kg [36].

Other important trepang collection areas during the eighteenth and nineteenth century were West-Papua (VII), Sumbawa (VIII), Timor (IX) and Selayar (X), as well as two regions in Northern Australia, namely Marege (XI) and Kayu Jawa (XII) (see Figure 1). The Spermonde Archipelago remained an important collection site over time. In the mid-nineteenth century, the best quality of trepang, the so-called *trepang pasir*, was obtained from sandy banks around the island of Kuding Aring, today known as Kodingareng [39]. In 1921, Spermonde was still named as one of the richest trepang fishing grounds [40].

The commercial fishing reached a peak in the mid-eighteenth century. Around 1850, the annual export of dried trepang from the Kei Islands was estimated at 36 tons, between 600,000 and 1,200,200 specimens [41]. Yearly shipping from Makassar was between 490 and 550 tons [42].

It is difficult to determine how many species of trepang were fished. Koningsberger [43] listed 112 different local names, several of them describing the same species. Table S1 lists a range of commercially relevant trepang species mentioned in the literature between 1812 and 2004. The references cited in the supplementary tables are listed in Supplementary Text S1.

While the species definitely played a role in determining the locally distinguished variety, the size of the animal and the skills used in processing were also important [5]. Thus, diverse colloquial names referred to the same species, whose value could differ widely. In 1820, Crawford described: “In the market of Makassar, the greatest staple of this fishery, not less than thirty varieties are distinguished … the trepang is one in which no stranger can embark with any safety.” [44:442]. The most expensive was the already mentioned *trepang pasir* from the Spermonde Archipelago near Makassar. Part of its high quality was due to the skilful way in which the product was prepared by the Bajau there [8]. According to VOC sources, in the second half of the 18^th^ century one *pikul* of this variety was worth 77.5 rixdollars, while the cheapest kind (black trepang) sold for 10 rixdollars. At the same time, a *pikul* of rice cost about 1.5 rixdollars [38].

### Off to new territories–the voyage to Marege

The most fascinating and also comparatively well-known part of the Makassarese trepang fishery is its extension towards the northern coasts of Australia. Due to the extensive work of Charles Campbell Macknight, we have a relatively complete picture of the size and sophistication of what he has called “Australia's first modern industry” [5:1]. Because there is not much to add to his work, we will be rather brief here, and refer readers to his magnificent and detailed descriptions [5], [45].

The two major trepang fishing areas were Marege, also known as Arnhem Land and today a part of the Northern Territory, and Kayu Jawa, the so-called Kimberley Coast in Western Australia (see Figure 1). Lion [42] reported about activities at various locations at the east coast of Queensland, but this was apparently based on a misreading of earlier sources [46].

The British hydrogeographer Alexander Dalrymple was the first who, in 1768, reported about Bugis in Australia [47]. Some 30 years later, in February 1803, the navigator Matthew Flinders surveyed the north-eastern part of Arnhem Land. To him we owe him the first detailed description of trepanging in Australian waters:

“After clearing the narrow passage between Cape Wilberforce and Bromley's Isles, we followed the main coast to the S.W. having on the starboard hand some high and large islands, which closed in towards the coast a-head so as to make it doubtful weather there is any passage between them. Under the nearest island was perceived a canoe full of men; and in a sort of roadsted, at the south of the same island, there were six vessels covered over like hulks, as if laid up for the bad season.” [48:228–229]

As is turned out, the vessels came from Makassar. They were part of a fleet which each year-using the north-west monsoon starting in December–travelled along the coast of northern Australia in search for trepang. They were equipped with all necessities to build small camps close to the shore, where they also erected smokehouses for the preparation of trepang. The majorities of supplies were brought from Makassar; including tobacco and other commodities for Aboriginal people who were to a small extent also employed. Only firewood for smoke-drying was gathered from mangroves in the area.

Depending on the wind, about 10–15 days were needed for the 1,600 km from Makassar to Marege. Each year, up to 2000 men made the journey and were temporarily scattered in processing camps between the Cobourg Peninsula and the bottom of the Golf of Carpentaria. They mainly gathered *grey trepang*, also described as *chalk fish* or *white trepang*. While this variety was not the most valuable, it still fetched a reasonable price and was available in great quantities. According to contemporary sources, *trepang Marege* and *trepang Kayu Jawa* became distinguished varieties in the market, and the commercial success of the trepangers depended on their ability to transform the abundant *chalk fish* into *trepang Marege* or *trepang Kayu Jawa* [5]. When the wind changed in about April, the vessels returned to Makassar. Their average cargoes varied from 8.5 tons to more than 22 tons. Macknight [5] assumed a total production of some 350 tons per season in the first half of the nineteenth century, which seemed to have slightly decreased later.

According to Macknight [5], [27], Sutherland [7] and Boomgaard [2], the Makassan activities in Australia did last from at least 1750 to 1906–1907, when the South Australian government decided to stop trepanging in its waters. While among the official arguments put forth for the ban was the need to protect the Aboriginal population from the baneful influence of the Makassans, who imported spirits, it rather seems that anti-Asian sentiments played the main role: “The Makassans were prohibited because they were foreigners…” [5:124]. The political decision to curtail the Makassarese trepang industry in Australia did not only end the annual voyages between Makassar and Marege; it also stopped a cultural exchange that was certainly older than the earliest European contacts with the continent and that has been preserved in the form of rock and bark paintings left behind by 19^th^ and early 20^th^ century aboriginal Australians [49].

### Early signs of overfishing

When considering the amounts of sea cucumbers that were collected, it seems implausible that overfishing did not take place at least in some locations. As a matter of fact, a number of sources do contain descriptions which can be interpreted as more or less obvious signs of early overfishing.

Knaap and Sutherland [38] depict that when trepang became the most important commodity in Makassar at the end of the eighteenth century, a shift to more distant fishing grounds took place. This is supported by Klaehn, who in 1833 argued that the rise of trepang fishing at Australia's coasts might had been related to the fact that “the yield of the seas between the Moluccas and the Sunda Islands is not sufficient any more to satisfy the ever growing market in luxurious China” [50:90]. This continuously growing demand also becomes obvious from the amounts exported from Makassar shown in Table 1.

An explicit account of local overfishing was given by Bosscher [41], who remarked that in the islands of Aru, people would gather trepang at the same sand bank until the stock seemed to have been depleted. Polunin [33] reported about a general decline in trepang trade between 1840–1850, without mentioning the original sources of his information. Similarly, Vosmaer [51:180–184] describes a significant decline in trade in Makassar in the 1830s, but relates this to the politico-economic situation in the Archipelago at the time rather than ecological causes (see also [7]). Interestingly, the first steps to regulate the overexploitation of pearl-oysters were undertaken by the Dutch colonial government at the same time, indicating that depletion of marine resources actually was a concern at that time.

Shortly afterwards, a new harvesting technique was adopted along the Australian coasts: dredging. Searcy [52:28] provided the first description of this activity: “… twelve large dredging canoes coming down before the wind, and hauling the great trepang dredgers … The twelve canoes, which were almost in line, had their immense mat sails hoisted on the triangular mast, and were gliding through the rippling water … while just beyond the canoes were four proas [perahus, boats] at anchor, close to the beach, on which the Malay camp was formed.”.

Fishing for sea cucumbers became more effective. However, the more or less stable level of trepang exported from the region over a long time seems to confirm that overfishing, if it occurred, was probably a temporary problem of a limited area that was fished out for the year, but replenished before the next [5]. On the other hand, Macknight [5] also reported that the large quantities produced in the Queensland Coast and Torres Strait could not be maintained after the 1880s.

From the few sources available, it seems fair to assume that some overfishing did occur, but was restricted to certain locations and limited periods of time. Sufficient recruitment was obviously still possible, and species which occurred in greater depths were yet out of reach. The scattered information does not allow more detailed reasoning including an answer to the question if the changing spatial exploitation patterns were indeed a result of stock depletion or rather driven by competition. However, it should be kept in mind that historical overfishing could have gone largely unnoticed, especially for resources which had virtually no value outside the Asian markets.

### Industrialisation and recent overfishing

As discussed above, although considerable amounts of sea cucumbers have been harvested for centuries in some localities, ecological overfishing historically did not appear to pose a major threat since harvest was confined to comparatively shallow depths. Larger individuals occurring at greater depths possibly provided a pool of individuals that could replenish the population once it was depleted in shallow waters [43]. This situation changed towards the end of the 20^th^ century, when populations collapsed in many places because so many people took part in collecting. The age-old movement of certain groups of collectors from reef to reef accelerated as prices rose and stocks of certain species were quickly depleted in one area after another [12]. Also, new technology enabled far more efficient harvesting.

In the 1980s and 1990s, increasing demand from China and other parts of the world revived the trepang fishing activities [12] (see Table S2 in the supplementary material). Collectors in the Spermonde Archipelago began to make use of compressor diving to reach ever greater depths [53]. Consequently, a rapid decline in sea cucumbers was observed. While large specimens were still common on sandy bottoms deeper than 20 m in the 1980s, several species were rigorously depleted only a decade later [54]. By the end of the century, Massin [55:130] reported that “some reefs off Ujung Pandang [Makassar] have nearly been stripped from the largest and commercially important species such as *Holothuria nobilis*, *H. scabra*, *Stichopus* spp., *Thelenota* spp., and *Bohadschia* spp.”. Nowadays, most of the fishermen in Spermonde report that they travel large distances, e.g. to the coasts of Kalimantan, to collect trepang (unpublished data from own interviews in June and November 2009).

A study recently published by FAO [25] shows a similar situation for the major trepang areas worldwide, all of which are under intense harvesting pressure. With most of the more valuable species fully exploited or overexploited, fisheries have moved from low quantity-high value to high quantities-low value ventures, and also evolved from single-species to multispecies fisheries. The report also notes that “… sea cucumber fishing is not a traditional activity…” [25:6], and reasons that overfishing is related to the recent strong dependency of many coastal communities on trepang as an alternative income source. This is certainly not true for the Makassan trepanging activities, which look back to a long tradition of at least 300 years. The report does show, however, that the ever increasing demand in the twentieth century led to an expansion of sea cucumber fishing into virtually all suitable areas, and also enabled a far greater number of people to participate than those traditionally being involved.

### Potential effects on ecosystems

While the social systems linked to trepang fishing and trade have intensively been researched, the ecological effects of removing a large number of bioturbating organisms from shallow tropical ecosystems for the most part are still unknown [56]. A study on the recovery of *H. nobilis*, a highly sought-after species, showed that overfished stocks may take decades to recover [57], and that ecosystem impacts therefore are likely to be long-lasting. Model calculations have shown that the natural population of two sea cucumber species on a reef flat in the Great Barrier Reef potentially could rework the upper 5 mm of the sediment in the area within one year, thereby recycling nutrients within the sediment [58]. Thus, they play an important role in controlling the benthic microenvironment [59]–[60]. A healthy sediment community is important for the general functioning of coral reef ecosystems, as it has been shown that up to 50% of the net fixed carbon released by corals in the form of mucus can be retrieved by benthic recycling, thus supporting the characteristic high productivity of coral reefs [61].

In 1825, Kolff gave the following description from the Tanimbar Islands: “… we… passed through a number of small islands … and trepang lay on the banks in the greatest abundance.” [13:268]. In 1999, a survey in the Indo-Pacific revealed that four of the once most common species were totally absent from 39% of the surveyed reefs [62]. While more information is urgently needed, it seems highly unlikely that the depletion of a species that used to be a basic component of coral reef ecosystems should have no effects on the structure and functioning of these systems.

### Comparison with other marine resources and management implications

Certain regions in maritime Southeast Asia still largely depend on resources taken from the sea. Besides traditionally harvested commodities such as pearls, mother-of-pearl or shark fins, a number of new products have entered the market over the last decades, such as live food fish or ornamentals. While the exploitation of sea cucumbers is quite unique in its magnitude and in the role which certain ethnicities used to play in the trade, it also shares a number of similarities with more recent exploitation patterns of other resources.

A common feature is their exclusive destination for markets outside the country of origin. Thus, utilization changes according to international demands, and the goods flow through a shifting hierarchy of middlemen and other traders.

In regions such as South Sulawesi, the role of patrons or *punggawas* is central in the market chain. Patrons react to market signals by providing their clients with credits and/or the necessary equipment for fishing activities. Primary resource collectors can therefore hardly choose what to fish. They are bound to their *punggawa* by debts and have to adapt their exploitation strategies accordingly. While *punggawas* specialize in certain commodities, their clients do so as well. This has led to spatially differing exploitation patterns between locations: In the Spermonde Archipelago, some islands are known for their live food fishing activities, while others utilize ornamental fish and corals or trepang (own observations).

A pattern common to marine resource exploitation is the spatially expanding depletion of harvested species known as the “roving bandit syndrome”. Roving bandits profit from the *de facto* open access nature of marine resources in many areas, where property rights are not defined or secured. Such roving collectors are thought to have no incentive for the local conservation of resources. The proposed remedy is the so-called “stationary bandit”, which simply means that fishermen stay in one place. As they are dependent on the future existence of their local resources, stationary users are hypothesized to have a vested interest in the maintenance of these resources [63]. However, in the marine realm, there are examples where mobility is used as a strategy to conserve resources: regular travels by Bajau to areas off their all-day fishing grounds were also a traditional strategy to avoid local overexploitation [64]. Ending such practises would therefore even increase overexploitation in some areas. This strengthens the demand for spatial and temporal flexibility in the management of marine resources. More flexible regulations (may) help to include local communities in conservation measures, which in turn increases their effectiveness [65].

## Discussion

For centuries, trepang has played an important role in the economic development of maritime Southeast Asia. It laid the foundations for a complex web of relationships, integrating scattered seafaring populations into long distance trade, and intensifying the commercial possibilities of outlying regions.

While trepang fishing shares a number of similarities with the exploitation of other marine resources, such as (1) a strong influence of international markets, (2) the role of patron-client-relationships and (3) the roving bandit syndrome, there is also a major difference: its longevity. For 300 years, trepang remained a major export commodity in island Southeast Asia. Fishing for live food fish such as grouper has a history of less than 30 years. After half of that time, some waters including Riau and the Spermonde Archipelago were already fished out. In order to obtain reasonable catches, divers based in these islands must now roam further away, e.g., to Take Bone Rate Atoll and through the Moluccas and Raja Ampat in West Papua. Additionally, fashion is extremely important and plays a major role in determining a fish's desirability. Like any fashion-driven preference, preferred species of live reef fish tend to change with time [21]. Thus, when a species is fished out, or simply drops in demand, new sorts will be targeted. In a way, the modern form of trepang fishery, utilizing compressor diving and reaching to ever-increasing depths, is a novel activity more similar to other emerging marine fisheries, and should be distinguished from the century-old practise of collection in shallow waters.

Another major difference is the existence of certain management structures. Trepang fishing in some areas led to the development of property rights which determined the right to capture sea cucumbers. Harvest restrictions were implemented by temporal closures of collection areas, the so-called *sasi teripang*, and local overexploitation was avoided by travelling to other areas. Such institutions are completely absent for more recently exploited resources, and also for trepanging in its modern way. Alternative management forms have to be created, such as pro-active management plans beyond the periphery of presently exploited areas [66].

Thus, understanding the similarities and differences between historical and recent exploitation of marine resources, as is currently being attempted by several authors in the frame of the History of Marine Animal Populations (HMAP) project, constitutes an important step towards finding sustainable solutions for the emerging problems.

## Materials and Methods

This article draws on an extensive analysis of historical documents and a number of secondary sources. Research for this article was initiated by a systematic search of digitalized documents available in the online repository of the Royal Netherlands Institute of Southeast Asian and Caribbean Studies (KITLV) in Leiden, The Netherlands and through google books. Data was then augmented by original documents derived mainly from two archives: the KITLV in Leiden, and the National Archives of the Republic of Indonesia (ANRI) in Jakarta, Indonesia. The historical information was complemented with empirical findings from field visits to Makassar and the Spermonde Archipelago between August 2008 and January 2010.

## Supporting Information

Table S1 Commercially relevant trepang varieties with their local and scientific names.
(0.13 MB DOC)

Table S2 Makassarese trepang export and production from 1975–2009.
(0.07 MB DOC)

Text S1 References for Tables S1–S2.
(0.03 MB DOC)

## References

[1] Wallace AR (1869). The Malay Archipelago: The Land of the Orang-utan, and the Bird of Paradise.. A Narrative of Travel, With Studies of Man and Nature.

[2] Boomgaard P, Boomgaard P, Henley D (2005). Resources and people of the sea in and around the Indonesian Archipelago, 900–1900.. Muddied Waters. Historical and Contemporary Perspectives on Management of Forest and Fisheries in Island Southeast Asia.

[3] Sutherland H (2004). The Sulu Zone Revisited.. J Southeast Asia St.

[4] Taylor JG (2003). Indonesia: People and Histories..

[5] Macknight CC (1976). The Voyage to Marege.. Macassan trepangers in northern Australia.

[6] Dai Y, Wu DYH, Cheung SCH (2002). Food Culture and Overseas Trade: The Trepang between China and Southeast Asia during the Qing Dynasty.. The globalization of Chinese food.

[7] Sutherland H (2000). Trepang and Wangkang. The China trade of eighteenth- century Makassar c. 1720s-1840.. Bijdr Taal-Land-V.

[8] Sopher DE (1965). The Sea Nomads: A study based on the literature of the maritime boat people of Southeast Asia..

[9] Earl GW (1837). The Eastern Seas or Voyages and Adventures in the Indian Archipelago in 1832-33-34..

[10] Leirissa RZ (1993). The Structure of Makassar- Bugis Trade in Premodern Moluccas.. Rev Indon Malay Affairs.

[11] Reid A (1999). Charting the Shape of early Modern Southeast Asia..

[12] Butcher JG (2004). The Closing of the Frontier: A History of the Marine Fisheries of Southeast Asia c.. editors.

[13] Kolff DH (1840). Voyages of the Dutch brig of War Dourga, through the southern and little-known parts of the Moluccan Archipelago, and along the previously unknown southern coast of new guinea..

[14] Andaya BW (2006). The Flaming Womb: repositioning women in early modern Southeast Asia:.

[15] Pelras C (2000). Patron-client ties among the Bugis and Makassarese of South Sulawesi.. Bijdr Taal-Land-V.

[16] Anonymous (1854). De nijverheid op Celebes.. Tijdschrift voor Nederlandsch-Indië.

[17] Sutherland H, Lapian AB (2000). Money in Makassar: Credit and debt in an eighteenth century VOC settlement.. Arung Samudera: persembahan memperingati sembilan windu.

[18] Meereboer M-T, Robinson K, Paeni M (1998). Fishing for credit: Patronage and debt relations in the Spermonde Archipelago, Indonesia.. Living through histories. Culture, history and social life in South-Sulawesi.

[19] Berkes F, Hughes TP, Steneck RS, Wilson JA, Bellwood DR (2006). Globalization, Roving Bandits, and Marine Resources.. Science.

[20] Huitric M (2003). Lobster and conch fisheries of Belize: A history of sequential exploitation.. Ecol Soc.

[21] Johannes RE, Riepen M (1995). Environmental, economic and social implications of the live reef fish trade in Asia and the Western Pacific..

[22] Reksodihardjo-Lilley G, Lilley R (2007). Towards a sustainable marine aquarium trade: An Indonesian perspective.. SPC Live Reef Fish Inf Bull.

[23] Cadigan ST, Hutchings JA, Holm P, Smith TD, Starkey DJ (2001). Nineteenth-Century Expansion of the Newfoundland Fishery for Atlantic Cod: An Exploration of Underlying Causes.. The Exploited Seas: New Directions for Marine Environmental History.

[24] Jackson JBC, Kirby MX, Berger WH, Bjorndal KA, Botsford LW (2001). Historical Overfishing and the Recent Collapse of Coastal Ecosystems.. Science.

[25] Toral-Granda V, Lovatelli A, Vasconellos M (2008). Sea cucumbers: A Global Review of Fisheries and Trade..

[26] Hamel J-F, Conand C, Pawson DL, Mercier A (2001). The Sea Cucumber *Holothuria scabra* (Holothuroidea: Echinodermata): Its Biology and Exploitation as Beche-de-Mer.. Adv Mar Biol.

[27] Macknight CC (1973). The nature of early maritime trade: some points of analogy from the eastern part of the Indonesian archipelago.. World Archaeol.

[28] Bickmore AS (1868). Travels in the East Indian Archipelago..

[29] Earl GW (1850). The trading ports of the Indian Archipelago.. Journal of the Indian Archipelago and East Asia.

[30] Osseweijer M, Ellen R, Parkes P, Bicker P (2000). ‘We wander in our ancestors' yard’: Sea cucumber gathering in Aru, Eastern Indonesia.. Indigenous Environmental Knowledge and its Transformations: Critical Anthropological Perspectives.

[31] Butcher JG (2004). The Closing of the Frontier.. A History of the Marine Fisheries of Southeast-Asia c. 1850-2000.

[32] Zerner C (1994). Through a green lens: The construction of customary environmental law in Indonesia's Maluku Islands.. Law Soc Rev.

[33] Polunin NVC (1983). The Marine Resources of Indonesia.. Oceanogr Mar Biol.

[34] Zerner C, Grove RH, Damodaran V, Sangwan S (1998). Men, Molluscs and the Marine Environment in the Maluku Islands: Imagining Customary Law and Institutions in Eastern Indonesia 1870–1992.. Nature and the Orient. The Environmental History of South and Southeast Asia.

[35] Radermacher JCM (1786). Korte Beschrijving van het eiland Celebes, en de eilanden Floris, Sumbauwa, Lombok en Baly.. Verhandelingen van het Bataviaasche Genootschap der Kunsten en Weetenschappen.

[36] Kelly P (1835). The Universal Cambist and Commercial Instructor..

[37] Sutherland H, Quarles van Ufford P, Schoffeleers M (1988). Power, Trade and Islam in the Eastern Archipelagos, 1700–1850.. Religion & Development: Towards an integrated approach.

[38] Knaap G, Sutherland H (2004). Monsoon Traders: Ships, Skippers and Commodities in Eighteenth-Century Makassar..

[39] Anonymous (1842). Der südliche Molukken-Archipel. Die Aru-Inseln. Dritter Abschnitt. In: Widenmann G, Das Ausland.. Ein Tagblatt für Kunde des geistigen und sittlichen Lebens der Völker.

[40] Stibbe DG (1921). Encyclopaedie van Nederlandsch-Indie.. Part 4: Sooemb-Z.

[41] Bosscher C (1855). Bijdrage tot de kennis van de Keij-eilanden.. Tijdschrift voor Indische Taal-, Land- en Volkenkunde IV.

[42] Lion HJ (1855). De Tripang-visscherij.. Tijdschrift voor Niederlandsch Indie.

[43] Koningsberger JC (1904). Tripang en Tripangvisscherij in Nederlandsch- Indie..

[44] Crawford J (1820). History of the Indian Archipelago..

[45] Macknight CC (1969). The Farthest Coast..

[46] Cense AA (1952). Makassaars-Boeginese Prauwvaart op Noord-Australie.. Bijdr Taal-Land-V.

[47] Dalrymple A (1771). A plan for extending the commerce of this kingdom and of the East-India-Company.. J Nourse and T Payne.

[48] Flinders M (1814). A voyage to Terra Australis; undertaken for the purpose of completing the discovery of that vast country, and prosecuted in the years 1801, 1802, and 1803..

[49] Burningham N (1994). Aboriginal Nautical Art: a record of the Macassan and the pearling industry in Northern Australia.. The Great Circle.

[50] Klaehn, Ersch FG, Gruber FG (1833). Holothurienbank.. Allgemeine Encyklopadie der Wissenschaften und Künste.

[51] Vosmaer JN (1839). Korte beschrijving van het zuid-oostelijk schiereiland van Celebes, in het bijzonder van de Vosmaers-Baai of van Kendari: verrijkt met eenige berigten omtrent den stam der Orang Badjos en meer andere aantekeningen.. Verhandelingen van het Bataviaasch Genootschap van Kunsten en Wetenschappen.

[52] Searcy A (1909). In Australian Tropics..

[53] Erdmann M (1995). An ABC Guide to Coral Reef Fisheries in Southwest Sulawesi, Indonesia.. Naga, The ICLARM Quarterly.

[54] Hoeksema BW, Visser LE (2004). Biodiversity and the Natural Resource Management of Coral Reefs in Southeast Asia.. Challenging Coasts Transdisziplinary excursions into integrated coastal zone development.

[55] Massin C (1999). Reef-dwelling Holothuroidea (Echinodermata) of the Spermonde Archipelago (South-West Sulawesi, Indonesia)..

[56] Massin C, Jangoux M, Lawrence JM (1982). Effects of feeding on the environment: Holothuroidea.. Echinoderm Nutrition.

[57] Uthicke S, Welch D, Benzie JAH (2004). Slow growth and lack of recovery in overfished holothurians on the Great Barrier Reef: Evidence from DNA fingerprints and repeated large-scale surveys.. Cons Biol.

[58] Uthicke S (1999). Bioturbation and impact of feeding activity of *Holothuria* (*Halodeima*) *atra* and *Stichopus chloronotus*, two sediment feeding holothurians, at Lizard Island, Great Barrier Reef.. Bull Mar Sci.

[59] Uthicke S, Klumpp S (1998). Microphytobenthos community production at a near-shore coral reef: seasonal variation and response to ammonium recycled by holothurians.. Mar Ecol-Prog Ser.

[60] Michio K, Kengo K, Yasunori K, Hitoshi M, Takayuki Y (2003). Effects of deposit feeder *Stichopus japonicus* on algal bloom and organic matter contents of bottom sediments of the enclosed sea.. Mar Poll Bull.

[61] Wild C, Huettel M, Klueter A, Kremb SG, Rasheed MYM (2004). Coral mucus functions as an energy carrier and particle trap in the reef ecosystem.. Nature.

[62] Hodgson G (1999). A global assessment of human effects on coral reefs.. Mar Poll Bull.

[63] Olson M (2000). Power and Prosperity..

[64] Lowe C (2006). Wild profusion: biodiversity conservation in an Indonesian archipelago..

[65] Ferse SCA, Mánez Costa M, Schwerdtner Mánez K, Adhuri DS, Glaser M (2010). Allies, not aliens - increasing the role of local communities in MPA implementation..

[66] Scales H, Balmford A, Liu M, Sadovy I, Manica A (2006). Keeping bandits at bay?. Science.

[67] Anonymous (1882). Zeevisscherijen langs de kusten der eilanden van Nederlandsch–Indie. V. Celebes.. Tijdschrift voor Nijverheid en Landbouw en Niederlandsch-Indie.

