# Supplementary material for: The History of Makassan Trepang Fishing and Trade
Source: PLoS One. 2010 Jun 29;5(6):e11346. doi: 10.1371/journal.pone.0011346 (PMC2894049; doi:10.1371/journal.pone.0011346)
Supplement: Table S1 — Commercially relevant trepang varieties with their local and scientific names. (0.13 MB DOC) [file pone.0011346.s001.doc]

Table S1: Commercially relevant trepang varieties with their local and scientific names.

| **Local name** | **Origin** | **Latin name** | **1700-1750** | **1750-1800** | **1800-1850** | **1850-1900** | **1900-1950** | **1950-2000** | **>2000** |
| --- | --- | --- | --- | --- | --- | --- | --- | --- | --- |
| Ballang ulu | makassarese | *Actinopyga mauritiana* |  |  |  |  |  | [9]^a^, [10]^a,b^ | [11]^a,b^ |
| Bangkuli, Buang kulit | indon. (sheds skin) | *Holothuria scabra* |  |  | [3,4,5]^a^ | [7]^a^ |  | [9]^a^, [10]^a,b^ | [11]^a,b^ |
| Batu | indon. (stone) | *Holothuria nobilis* | [1]^a^ | [1]^a^ | [2,3,4,5]^a^ | [7]^a^ | [8]^a,b^ | [10]^a,b^ | [11]^a,b^ |
| Batuna | ? | *Holothuria hilla* |  |  |  |  | [8]^a,b^ |  |  |
| Bilala, Bilalo | ? | *Actinopyga echinites*? |  |  | [5]^a^ |  |  |  | [12]^a,b^ |
| Binti | makas. (spotted) | *Bohadschia argus* |  |  |  |  |  | [10]^a,b^ | [11]^a,b^ |
| Bissawa | makassarese | *Holothuria fuscogilva* |  |  |  |  |  | [10]^a,b^ | [11]^a,b^ |
| Cari-cari | indon. (searching) | ? |  |  |  | [7]^a^ |  |  |  |
| Cera | ? | *H. leucospilota, H. atra, H. edulis* |  |  |  | [7]^a^ | [8]^a,b^ | [10]^a,b^ |  |
| Donga | possibly: dongak, indon. (raised) | *Thelenota anax, also Paersonothuria graeffi*? |  |  | [3,4,5]^a^ | [7]^a^ |  | [9]^a^, [10]^a,b^ |  |
| Gama | possibly: gamat, indon. (generic for sea cucumber) | *Stichopus variegatus, Bohadschia vitiensis*? |  | [1]^a^ | [3,4,5]^a^ | [7]^a^ |  | [10]^a,b^ | [12]^a,b^ |
| Gama bati | makas. (batik sea cucumber) | *Bohadschia argus*?, *Paersonothuria graeffi*?, *Thelenota rubrolineata*? |  |  | [4]^a^ |  |  | [10]^a,b^? |  |
| Gama gemuk | indon. (fat sea cucumber) | *Bohadschia argus*?, *Paersonothuria graeffi*? |  |  | [4]^a^ |  |  |  |  |
| Gatta | makassarese | *Bohadschia vitiensis* |  |  |  |  |  | [9]^a^, [10]^a,b^ | [11]^a,b^ |
| Gosok | indon. (paste, powder) | *H. scabra* |  |  | [4]^a^ |  |  |  |  |
| Hitam | indon. (black) | *Actinopyga* sp. (*A. miliaris, A. lecanora*) | [1]^a^ | [1]^a^ | [3,4,5]^a^ |  |  | [9]^a^ |  |
| Japang, Japon, Japun | indon. (japanese) | *Stichopus chloronotus, Holothuria leucospilota*? |  | [1]^a^ | [3,4]^a^ | [7]^a^ | [8]^a,b^ | [10]^a,b^ |  |
| Kacang goreng | indon. (roasted peanut) | *Stichopus horrens* |  |  | [3,4,5]^a^ | [7]^a^ |  |  |  |
| Kassi | makas. (sand) | *Holothuria scabra, also Actinopyga echinites, A. miliaris* |  |  | [4]^a^ | [7]^a^ |  | [10]^a,b^ | [11]^a,b^ |
| Kassut, Kasu | ? | ? |  |  | [4]^a^ | [7]^a^ |  |  |  |
| Kawas, Kawasa, Kuwas | ? | *Bohadschia marmorata* |  |  | [3,4,5]^a^ | [7]^a^ |  |  |  |
| Kayu Java | place name (Australia) | *H. nobilis*? |  |  | [3,4,5]^a^ |  |  |  |  |
| Kebo | makas. (white) | *Holothuria rigida*? |  |  | [4]^a^ | [7]^a^ |  |  |  |
| Keling, Kling |  | *Holothuria leucospilota*, also *H. atra* |  |  |  |  | [8]^a,b^ |  | [12]^a,b^ |
| Koro | makassarese | *Holothuria fuscogilva* |  |  | [2,4,5]^a^ | [7]^a^ | [8]^a,b^ | [9]^a^ | [11]^a,b^ |
| Kossong | indon. (empty) | *Actinopyga caerulea*? |  |  | [5]^a^ |  |  |  |  |
| Kuning | indon. (yellow) | *Holothuria fuscopunctata*? |  | [1]^a^ | [4]^a^ |  |  | [10]^a,b^ |  |
| Kunyi, Kunyit | indon. (turmeric) | *Holothuria fuscopunctata* |  | [1]^a^ | [3,5]^a^ | [7]^a^ |  | [10]^a,b^ | [11]^a,b^ |
| Lada-lada | indon. (pepper grains) | ? |  |  |  | [7]^a^ |  |  |  |
| Leleng, Luleng | makas. (black) | *Actinopyga miliaris, A. lecanora*? |  |  | [4]^a^ | [7]^a^ |  |  |  |
| Marege | place name | *Holothuria leucospilota*? |  | [1]^a^ | [3,4,5]^a^ |  |  |  |  |
| Massee, Mosi, Mossee | ? | ? |  | [1]^a^ | [3,4,5]^a^ | [7]^a^ |  |  |  |
| Nanas | indon. (pineapple) | *Thelenota ananas* |  |  | [4,5]^a^ |  |  | [9]^a^, [10]^a,b^ |  |
| Olok-olok | makassarese | *Bohadschia vitiensis*, also *B. marmorata* |  |  |  |  |  | [9]^a^, [10]^a,b^ | [11,12]^a,b^ |
| Pandan, Pandang | makassarese | *Thelenota ananas* |  | [1]^a^ | [4,5]^a^ | [7]^a^ |  | [9]^a^, [10]^a,b^ | [11]^a,b^ |
| Pasir | indon. (sand) | *Holothuria scabra* |  | [1]^a^ | [4,5]^a^ | [6]^a^ |  | [10]^a,b^ | [12]^a,b^ |
| Patola | makassarese | *Bohadschia argus* |  |  |  |  |  | [10]^a,b^ | [11]^a,b^ |
| Puti | indon. (white) | *Holothuria rigida*? | [1]^a^ |  | [4,5]^a^ |  |  |  |  |
| Radja | indon. (king) | *Holothuria scabra* |  |  | [4]^a^ |  |  |  |  |
| Susu | indon. (milk) | *Holothuria fuscogilva*, also *H. nobilis, H. rigida*? |  |  | [4]^a^ |  |  | [10]^a,b^ | [11,12]^a,b^ |
| Taceritang | probably: tanceretang, madurese (to defecate) | ? |  |  | [3]^a^ |  |  |  |  |
| Tai Kongkong | makas. (dog excrement) | *Stichopus* sp. (TKK herder: *S. variegatus, S. hermanni*, TKK batu: small *S. hermanni*?, TKK gonrong*: S. horrens*) |  | [1]^a^ | [3,4,5]^a^ | [7]^a^ |  | [9]^a^, [10]^a,b^ | [11]^a,b^ |
| Talengko | makas. (anchor rope) | *H. leucospilota, H. coluber*? |  |  |  | [7]^a^ |  | [9]^a^? |  |
| Tundang | makas. (to sit) | ? |  |  | [3]^a^ |  |  |  |  |

^a^ cites Indonesian names, ^b^ cites Latin names
