# Supplementary material for: The History of Makassan Trepang Fishing and Trade
Source: PLoS One. 2010 Jun 29;5(6):e11346. doi: 10.1371/journal.pone.0011346 (PMC2894049; doi:10.1371/journal.pone.0011346)
Supplement: Table S2 — Makassarese trepang export and production from 1975–2009. (0.07 MB DOC) [file pone.0011346.s002.doc]

Table S2: Makassarese trepang export and production from 1975-2009.

| **Year** | **Export [tons]** | **Production [tons]** | **Source** |
| --- | --- | --- | --- |
| 1975 | 200^a^ |  | [13] |
| 1976 | 172.0 |  | [14] |
| 1977 | 150^a^ |  | [13] |
| 1978 | 139.45 |  | [14] |
| 1979 | 200^a^ |  | [13] |
| 1980 | 100^a^ | 1.6 | [13,15] |
| 1981 | 350^a^ |  | [13] |
| 1982 | 500^a^ |  | [13] |
| 1983 | 1,100^a^ |  | [13] |
| 1984 | 1,150^a^ |  | [13] |
| 1985 | 1,150^a^ |  | [13] |
| 1986 | 2,100^a^ | 4.0 | [13,15] |
| 1987 | 1,700^a^ | 6.2 | [13,15] |
| 1988 | 1,750^a^ |  | [13] |
| 1989 | 1,400^a^ | 78.1 | [13,15] |
| 1990 |  | 102.9 | [15] |
| 1991 |  | 10.5 | [15] |
| 1992 |  | 111.3 | [15] |
| 1993 |  | 271.8 | [15] |
| 1994 |  | 777.2 | [15] |
| 1995 |  | 412.1 | [15] |
| 1996 | 359 | 85.7 | [11,15] |
| 1997 | 853 | 224.5 | [11,15] |
| 1998 | 399 | 248.0 | [11,15] |
| 1999 | 192 | 246.6 | [11,15] |
| 2000 | 146 | 281.2 | [11,15] |
| 2001 | 457 | 326.9 | [11,15] |
| 2002 | 241 | 636.0 | [11,15] |
| 2003 |  | 586.6 | [15] |
| 2004 | n/a |  |  |
| 2005 |  | 944.4 | [15] |
| 2006 | 318.1 | 962.4 | [15,16] |
| 2007 | 96.3 | 734.0 | [15,16] |
| 2008 | 21.6 | 754.9 | [15,16] |
| 2009 | 30.5 |  | [16] |

^a^ accuracy of this data only to 5x10^4^ kg
