# Supplementary material for: The History of Makassan Trepang Fishing and Trade
Source: PLoS One. 2010 Jun 29;5(6):e11346. doi: 10.1371/journal.pone.0011346 (PMC2894049; doi:10.1371/journal.pone.0011346)
Supplement: Text S1 — References for Tables S1–S2. (0.03 MB DOC) [file pone.0011346.s003.doc]

**Works Cited**

1. Knaap G, Sutherland H (2004) Monsoon Traders: Ships, Skippers and Commodities in Eighteenth-Century Makassar. Leiden: KITLV Press. 269 p.

2. Flinders M (1814) A voyage to Terra Australis; undertaken for the purpose of completing the discovery of that vast country, and prosecuted in the years 1801, 1802, and 1803. London: G. & W. Nicol. 828 p.

3. Crawfurd J (1820) History of the Indian Archipelago. Edinburgh: Archibald Constable & Co. 520 p.

4. Vosmaer JN (1839) Korte beschrijving van het zuid-oostelijk schiereiland van Celebes, in het bijzonder van de Vosmaers-Baai of van Kendari : verrijkt met eenige berigten omtrent den stam der Orang Badjos en meer andere aantekeningen. Verhandelingen van het Bataviaasch Genootschap van Kunsten en Wetenschappen 17: 61-184.

5. Kolff DH (1840) Voyages of the Dutch brig of War Dourga, through the southern and little-known parts of the Moluccan Archipelago, and along the previously unknown southern coast of new guinea. London: James Madden & co. Leadenhall Street. 365 p.

6. van Doren JBJ (1856) Beknopte Beschrijving van Wahaai. Bijdr Taal-Land-V 4: 181-207.

7. Engelhard HED (1884) Mededeelingen over het Eiland Saleijer. Bijdr Taal-Land-V 32: 263-491.

8. Weber M (1906) Les Pays-Bas. Exposition Internationale d`Oceanographie des Peches maritimes et des Produits de la Mer a Marseille. 180 p.

9. Conand C, Tuwo A (1996) Commercial holothurians in South Sulawesi, Indonesia. Fisheries and mariculture. Bêche-de-mer Information Bulletin 8: 17-21.

10. Tuwo A, Conand C (1996) Commercial Holothurians in Southwest Sulawesi (preliminary observations). Torani Buletin Ilmu Kelautan 6: 130-134.

11. Tuwo A (2004) Status of sea cucumber fisheries and farming in Indonesia. In: Lovatelli A, Conand C, Purcell S, Uthicke S, Hamel J-F, et al., editors. Advances in sea cucumber aquaculture and management. Rome: FAO Fisheries Technical Paper 463. pp. 49-55.

12. Hartati ST, Wahyuni IS, Suprapto, Reswati E (2002) Perikanan teripang di perairan kepulauan Seribu. Jurnal Penelitian Perikanan Indonesia 8: 55-64.

13. Dinas Perdagangan dan Perindustrian (South Sulawesi Department of Trade and Industry), cited in Tuwo A, Nessa MN (1991) Beberapa aspek biologi teripang ekonomis penting. Torani 1: 1-20.

14. Dinas Perikanan (Fisheries Department) South Sulawesi Annual Report.

15. Dinas Perikanan (Fisheries Department) South Sulawesi Annual Fisheries Statistics.

16. Dinas Perdagangan dan Perindustrian (South Sulawesi Department of Trade and Industry).
